# Ten simple rules for hosting artists in a scientific lab

**DOI:** 10.1371/journal.pcbi.1008675

**Published:** 2021-02-25

**Authors:** Matthias C. Rillig, Karine Bonneval, Christian de Lutz, Johannes Lehmann, India Mansour, Regine Rapp, Saša Spačal, Vera Meyer

**Affiliations:** 1 Freie Universität Berlin, Institut für Biologie, Berlin, Germany; 2 Berlin-Brandenburg Institute of Advanced Biodiversity Research (BBIB), Berlin, Germany; 3 6 Chemin des Vignes, Pesselières, Jalognes, France; 4 Art Laboratory Berlin, Berlin, Germany; 5 Soil and Crop Sciences, College of Agriculture and Life Sciences, Cornell University, Ithaca, New York, United States of America; 6 Studio Agapea, Ljubljana, Slovenia; 7 Chair of Applied and Molecular Microbiology, Institute of Biotechnology, Technische Universität Berlin, Berlin, Germany; Dassault Systemes BIOVIA, UNITED STATES

## Introduction

Hosting an artist in a scientific lab is likely a new experience for many scientists in the natural and engineering sciences, and perhaps also for many artists, yet it can be a very beneficial experience for both parties [1]. “Art and science are in a tension that is most fruitful when these disciplines observe and penetrate each other and experience how much of the other they themselves still contain” [2]. During our science and art collaborations in the last years, we have learned what connects and what separates our disciplines, how different yet common our worlds of working and thinking are, and how stimulating such collaborations can be. Although scientists and artists belong to two different cultural worlds, many share research as a congruent method to explore and understand the world around us. Often, scientific and artistic work spaces are indistinguishable as they are full of equipment, materials, tools, and computers to run experiments and analyze data [3,4]. Science and art are fundamentally connected through their focus on creativity [5]. Also, both scientists and artists deliberately venture into the public realm in the spirit of Hannah Arendt: “Humanity is never won in loneliness and never by handing one’s work over to the public. Only if you take your life and person[ality] into the venture of the public realm, will you reach [humanity]” [6]. At the most fundamental level, science and art both try to understand the world around us and to guide society to recognize and solve problems. Artistic and scientific research may also have much more in common than one expects at first sight: They both involve years of schools and personal development, they both involve trial and error, and the sharing of results with different communities.

However, transdisciplinary cooperation requires openness, a willingness to take risks, the ability for self-reflection, respect, and esteem for the other culture as well as a lot of appreciative listening from both parties [7,8]. Our paper thus intends to serve as a practical guide for both, artists-in-residence and the hosting scientific lab to easier cross borders, to better collaborate, to better learn from each other, and to sustainably bridge the different cultures of science and the arts. Our discussion starts at the point where a decision for such an interaction has already taken place. Still wondering if this is for you? There is much to gain for both sides. For the scientists, for example, this interaction can be a source of new ideas and questions, offering new points of view. Some of us also felt that this interaction offered training in explaining research in clear, simple language, and provided opportunities for interfacing with the science-curious public in a curated context. For the artists, this can be about learning new tools, methods, and approaches and about the specific topics on which a lab works.

**Fig 1:**
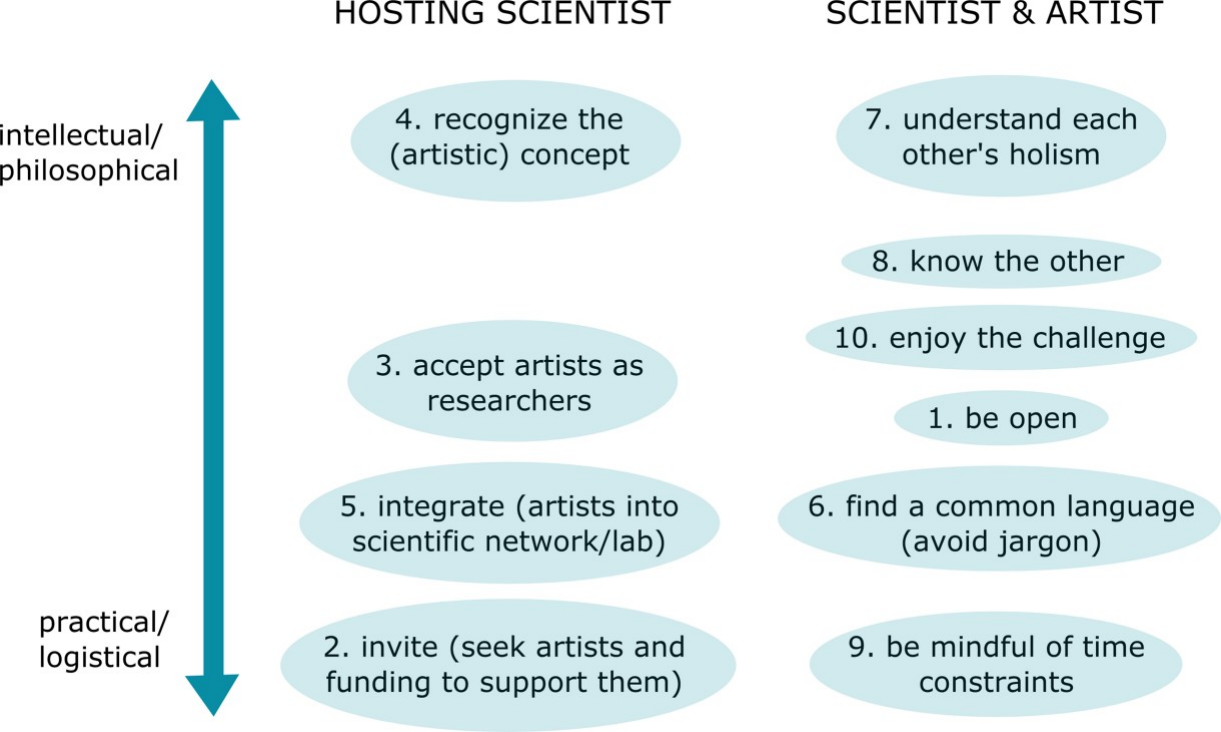
Summary of the 10 rules, arrayed from intellectual/philosophical to practical/logistical and divided into rules more applicable to the hosting science lab and rules relevant for both the hosting lab and the artist.

Some of the following 10 “rules” apply more to the artist, some more to the hosting science lab, and some to both (Fig 1).

### Rule 1: Be open

The basic mindset and attitude required for both scientists and artists is openness. Openness to new input, willingness to share ideas, and a sharing attitude. It is not at all required to have an artistic “inclination” on the part of the hosting scientist, but nothing can replace an open attitude. Be open to accept that research can be both: scientific research and artistic research (for a definition of artistic research, see [9]).

### Rule 2: Invite

Scientists may actively approach and invite artists to join their labs for a specific period of time. They should be aware that—although their own salaries are usually secured—the income of an artist is not secured. Hence, make sure to have a grant available that should cover the income of the artist and consumables for his or her artistic research.

Thus, seek out and visit art residencies and their show openings; make studio visits. Browse the internet and visit gallery shows and contact artists directly or get in touch with organizations that connect artists with scientists. Such organizations vary according to country and region. Some scientific organizations (e.g., CERN) have their own associated partners. In the EU, a number of organizations promote and support artists. Many of these have experience arranging collaborations: Examples are The Waag Society (NL), Kersnikova/Kapelica (SLO), BioArt Society (FI), Cultivamos Cultura and Ectopia (PT), Art Laboratory Berlin, and Schering Stiftung (D). Universities in many countries also support collaborations, and some have internal organizations that do so, for example, Metaphorest at Waseda University, Tokyo, SymbioticA at the University of Western Australia, and the MIT Media Lab.

Keep your lab’s websites up to date. Consider working with the artist when developing proposals for funding; this will ensure that sufficient funds are available and that the proposal is pitched in a way that works for the artist, but it will likely also be a much better proposal with much greater chance of funding the full project if it has a compelling broader impact module (if this is required for your national funding agency).

### Rule 3: Accept artists as researchers

An artist is not primarily in a scientific lab to perform outreach activities for lab’s research or illustrate and communicate scientific research results, even though this can also be an outcome. The artist is a researcher in the arts. Remember, while “the means” or the process may be the same, and the goals may also include to arrive at new insights, the product may take many different forms. The artist is doing research to produce an artwork, even if it may take the shape of sound art, a performance, or ephemeral objects. Some examples from our artist coauthors may help illustrate this point. Karine Bonneval researches the acoustic world surrounding plants. Her artworks bridge our perception and understanding of what plants are and how they create ecologies (https://www.karinebonneval.com/). Saša Spačal's research and installations create spaces for exploring symbiosis between life forms, often involving human interaction (https://www.agapea.si/en/).

### Rule 4: Recognize the concept

Scientists should not equate art projects with producing a painting to hang on the wall. Do not anticipate that an object will be created (even if in many or most cases it may be so). Focus on the concept, and allow the concept to be either directly related to the natural science/engineering research or also and specifically to creative practice (how do I develop an idea or a question?) as well as to sociopolitical dimensions.

### Rule 5: Integrate

The more effortlessly artists can become integrated in the scientific lab group and its routines, the better. For example, they could come to regular lab meetings, even give presentations there, or else participate in the technical discussions (certain topics are more appropriate than others). Ensure that the artist becomes embedded with his or her own place in the lab. Consider offering a lab tour to the artist at the beginning of the residency. All of this will make the artists more well-known in the lab group, and this is very important for the success of the artist’s residence, as it increases the number of potential interactions. Be open to learn as much as possible from the artist or the artistic practice in your science project as vice versa.

### Rule 6: Find common language

Different disciplines and surely artists and scientists use different languages. To understand each other, use or develop common language: explain terminology, both physical (what is a soil aggregate?) as well as conceptual (what does creativity mean?). Be aware that this process is probably the most time-consuming part of this joint endeavor. Even the same words can have different meanings, potentially leading to misunderstanding. Hence, sometimes, several rounds of explanation may be necessary to find a common language.

### Rule 7: Be curious about and understand each other’s holism

Most scientists in life sciences and engineering use holistic approaches to study, understand, and design (a)biotic systems. The biology of (micro)organisms are studied from the community, cellular, molecular, and physical levels with technologies and tools developed, e.g., in chemistry, biotechnology, computer, and material sciences. Hence, inter- and transdisciplinarity is a central working method for many scientists.

Artistic research often bridges different scientific disciplines with cutting-edge theory from the humanities (philosophy, cultural studies, ethics, and aesthetics) and social sciences (sociology and anthropology) creating both larger audiences and exchange of ideas across fields on topics such as the “anthropocene,” diversity loss, and antibiotic resistance, as well as questioning cultural viewpoints on the supposed divide between nature and (human) culture.

For both parties, it is helpful to be explicit about the other fields they draw upon in their work, making clear the respective “network of ideas” at play. This also requires awareness of our own intellectual context that we might normally take for granted when staying within our own respective “spheres” of science or the arts.

### Rule 8: Know the other

Interactions are effortless when the artist and scientist can communicate effectively and when the artist knows some background of the work done in the lab. Websites of many scientific groups introduce their scientific visions, approaches, technologies, and achievements. Scientific publications offer in-depth details and are often inspirational for artists. The more prepared artists are in knowledge and practice, the easier (and more successful) the residency will be.

Similarly, the scientist must not only know the media and oeuvre that the artist is working in, but also the progression and trajectory of concepts and questions. Familiarize yourself with the evolution of the artists thinking through the artists’ website, which is inspirational, too.

### Rule 9: Be mindful of time constraints on both sides

Scientists are typically busy with the day-to-day craziness of managing a lab, writing grants, and papers, so it is important to be mindful of these constraints and to fit into their schedule. This is best achieved by scheduling meetings that fit the academic structure, such as a 45-minute meeting or shorter, with specific goals.

Similarly, recognize that artists do not have unlimited time or may not be available when they are preparing shows or publications, writing grants, or are busy running their studios. Be flexible, when a meaningful interaction with artists is possible, and schedule meetings during those phases and days that provide opportunities to generate new insights.

### Rule 10: Enjoy the challenge

Merging science and the arts in one lab is not an easy endeavor neither for the artist nor the scientist and the scientists’ group. Getting appropriate funding, struggling with the difficulties in communication, and understanding of the different concepts and results is time-consuming, unpredictable, and uncontrollable (the latter can be a nightmare for some scientists). However, those who are willing to take on these challenges, who want to cooperate on eye level with the other, and who appreciate the other as companions will be rewarded with a tingling brain shower that can open new horizons in both artistic and scientific research. By joining scientific and artistic research, we could gain access to the wider complexity of a particular subject, inaccessible to one methodological approach alone.

We believe that when both parties consider these rules, they will be better prepared for the challenge, increasing the likelihood of a successful residency. With in-person residencies currently rendered challenging due to the pandemic, virtual (online) residencies are an option and may also be a good way to get acquainted during non-pandemic times.
